# The Effect of Scarpa Fascia Preservation in Lipo-Abdominoplasty on Abdominal Skin Perfusion Monitored by Infrared Thermography

**DOI:** 10.1007/s00266-025-05443-1

**Published:** 2025-12-09

**Authors:** Ahmed Ali Hassan, Shahad Majid Haqi, Ahmed Mamdouh Nafeh, Sameh Adel Desawy

**Affiliations:** 1 Plastic Surgery, Ain Shams University, Cairo, Egypt; 2 Plastic Surgery, Helwan University, Helwan, Egypt

**Keywords:** Lipoabdominoplasty, Infrared thermography

## Abstract

**Background:**

The safety of the lipoabdominoplasty is always a matter of concern specially the perfusion of the abdominal skin. Several anatomical studies of the anterior abdominal wall investigated the vascular supply of the skin after abdominoplasty. Different techniques were tried aiming to enhance the vascular perfusion of the distal abdominal skin. Scarpa fascia preservation reduced the likelihood formation of seroma. Recently, a single study reported that preservation of scarpa fascia underneath the abdominal flap enhances the perfusion and survival of the abdominal skin.

**Patients and Methods:**

In a Controlled randomized study, 20 patients underwent lipoabdominoplasty. 10 patients underwent standard lipoabdominoplasty (group I), while the other 10 patients underwent lipoabdominoplasty with scarpa fascia preservation. All the demographic data and preoperative conditions were consistent in both groups. Pre-, immediate postoperative and one-month postoperative measurement of distal skin temperature by Infrared Thermography (IRT) was recorded for both groups. The recorded temperature by IRT reflects the tissue perfusion and viability.

**Results:**

With a consistent demographic data and single surgical operating, Statistical analysis of the IRT measurements revealed a non-significant difference in patients of both groups.

**Conclusion:**

The preservation of scarpa fascia did not enhance the vascular perfusion of the distal skin after abdominoplasty. However, preservation of scarpa fascia may minimize the formation of postoperative seroma due to lymphatic drainage and a decreased dead space.

**Level of Evidence I:**

This journal requires that authors assign a level of evidence to each article. For a full description of these Evidence-Based Medicine ratings, please refer to the Table of Contents or the online Instructions to Authors www.springer.com/00266.

## Introduction

Abdominoplasty represented one of the most popular and commonly performed aesthetic surgical procedures [1, 2]. The increase in the procedure necessitates a need to decrease its likelihood local complications, of which the distal skin ischemia and necrosis is the most serious [3–5]. The combination of liposuction with abdominoplasty has been a matter of continuous discussion because the increased reports of the liposuction-induced impingement on the vascular supply of the resulting abdominal skin [6–8]. Matarasso, in 1991 [9] first described four regions that could be safely treated by liposuction when performing an abdominoplasty. He recommended limited and cautious liposuction when combined with a full abdominoplasty. However, he reported higher risk of flap necrosis when extensive flap undermining and liposuction was combined [10]. Along the history, several studies reported different modifications to achieve a safe lipoabdominoplasty [11–16]. Scarpa fascia preservation was first described by Le Louarn in 1996 [17] to enhance the lymphatic drainage and decrease the surgical dead space to minimize the postoperative seroma. The technique was adopted by others to investigate the reliability of scarpa fascia preservation [18–21].

Scarpa fascia preservation in lipoabdominoplasty reported good results in decrease the incidence of postoperative seroma and minimized the duration of the postoperative drain [21–24]. However, a single study has investigated the effect of scarpa fascia preservation on the enhancement of the distal abdominal skin perfusion [25]. The study reported better distal skin perfusion when scarpa fascia is preserved. However, the technique needs further investigation to confirm this result and as well to justify the result. Infrared thermography (IRT) is an imaging modality that allows the mapping and recording of body surface temperature, showing the physiology of blood microcirculation and providing indirect information on tissue perfusion [26, 27]. Infrared thermography is a precise, fast, contactless, portable, non-invasive, and non-ionizing tool and, therefore, safe for the patient and operator, with reduced costs. It is a reliable imaging tool used in esthetic and reconstructive surgery with similar accuracy to other invasive imaging techniques [28–30]. It is also a reliable tool for evaluating abdominal skin perfusion after abdominoplasty. This study was designed to investigate the effect of preserving the scarpa fascia in lipoabdominoplasty on the perfusion of abdominal skin monitored by non-invasive portable infrared thermography camera. However, the current study investigated the effect of preserving scarpa fascia on the vascularity of distal skin after lipoabdominoplasty not the seroma.

## Patients

This randomized controlled study included 20 women underwent abdominoplasty to treat abdominal skin redundancy and variable degrees of abdominal wall obesity. The study was performed in the department of plastic surgery, Ain Shams University Hospital, Egypt, Cairo, from January 2024 to the end of January 2025. The study was approved from the ethical committee of the faculty of medicine, Ain Shams University. Informed consent was signed by all patients enrolled in the study after introducing and explaining all the steps of the procedures including the potential complications and risk that might occur. The patients were randomly recruited into two groups; Group I: Standard lipoabdominoplasty without preservation of the scarpa fascia, Group II: Scarpa fascia preservation abdominoplasty. The study included women with an age ranged from 25 to 45 years, BMI ranged from 28 to 35 Kg/m^2^, Patient who have had fatty abdominal skin and flanks along with lower abdominal skin redundancy. All patients with a diagnosed abdominal wall hernias, previous abdominoplasty, previous abdominal liposuction, diabetes mellites, uncontrolled hypertension, and/ or smokers were excluded from the study. All patients were subjected to a preoperative clinical examination, laboratory investigation, and ultrasound of the abdominal wall. All patients were operated by the senior author.

## Methods

### Surgical Procedure

The procedure was carried out while the patient was lying supine. All patients received general anesthesia with endotracheal intubation. 1 gram of cephalexin was given intravenous after induction of anesthesia. Intermittent pressure pneumatic garment was applied on both feet and legs. Urinary catheter was inserted. Scrubbing of the surgical site was including the abdominal skin from the inframammary fold to the upper thighs. Sterile towels were applied exposing the abdominal skin from the upper thighs and mons pubis to the inframammary fold. A solution of adrenalin/saline 1/500000 was injected into the subcutaneous fat of the flanks and supraumbilical areas. Super wet technique (2:1 ratio) was used. Liposuction of both flanks and supraumbilical areas was carried out. A 4 mm liposuction cannula was used to lipoaspirate both flanks while a 3 mm cannula was used for supraumbilical and subcostal areas. Lower abdominoplasty incision was carried out reaching to scarpa fascia identified by its white glistening. Dissection was carried out at a supra scarpa plane until reaching a 5–7 cm above the lower abdominoplasty incision and then, the dissection was changed to a supra rectus plane until reaching the xiphisternum. (in case of standard abdominoplasty, the dissection was started supra rectus plane from the start). Separation of umbilicus and plication of recti was done using two layers of repair by 1/0 proline sutures. The skin of the abdomen was pulled down using two clamps and the proposed line of upper incision was marked. Resection of the excess skin was carried out and temporarily fixed to the lower flap by silk suture 1/0. The new site of the umbilicus was determined which was marked just opposite to the separated umbilicus. The umbilicus was relocated to its new site and fixed by subcutaneous 4/0 Vicryl and interrupted 4/0 proline sutures. The temporary silk sutures were removed and the scarpa fascia was tackled to the underneath of the abdominal flap by absorbable sutures (Vicryl 2/0). Suction drains were inserted underneath the abdominal flaps on both sides, the ports were exiting from the upper mons pubis. Closure of wounds was carried out, subcutaneous layer by 2/0 Vicryl and intradermal continuous suture by Monocryl 3/0. Dressing was applied over the wounds and pressure garment was applied.

## Postoperative Care

Postoperatively, all patients received 1 gm ceftriaxone intravenous every 12 hours, Clexane 40 IU every 12 hours for 5 days. Analgesics and antiedema drugs were given for 2 days. The first dressing was done after 3 days, and suction drains were removed when the volume of serous fluid of less than 30 cc was collected over 24 hours (average 2.5 days). Pressure garment was kept for 2 months.

### Infrared Thermography Assessment

Infrared thermography (IRT) measurements were taken at three fixed occasions, preoperative dynamic thermography (the day before surgery), intraoperative static thermography when the surgery was accomplished, and again the dynamic thermography at one-month postoperative. The measurements were taken at three fixed points, *R*1, *R*2 and *R*3). *R*1 and *R*2 areas were located 10 cm from the midline at both sides over the proposed line of lower abdominal incision, and *R*3 was located centrally at the midline just at the proposed lower abdominoplasty incision (most distal area of abdominal skin).

**Fig. 1:**
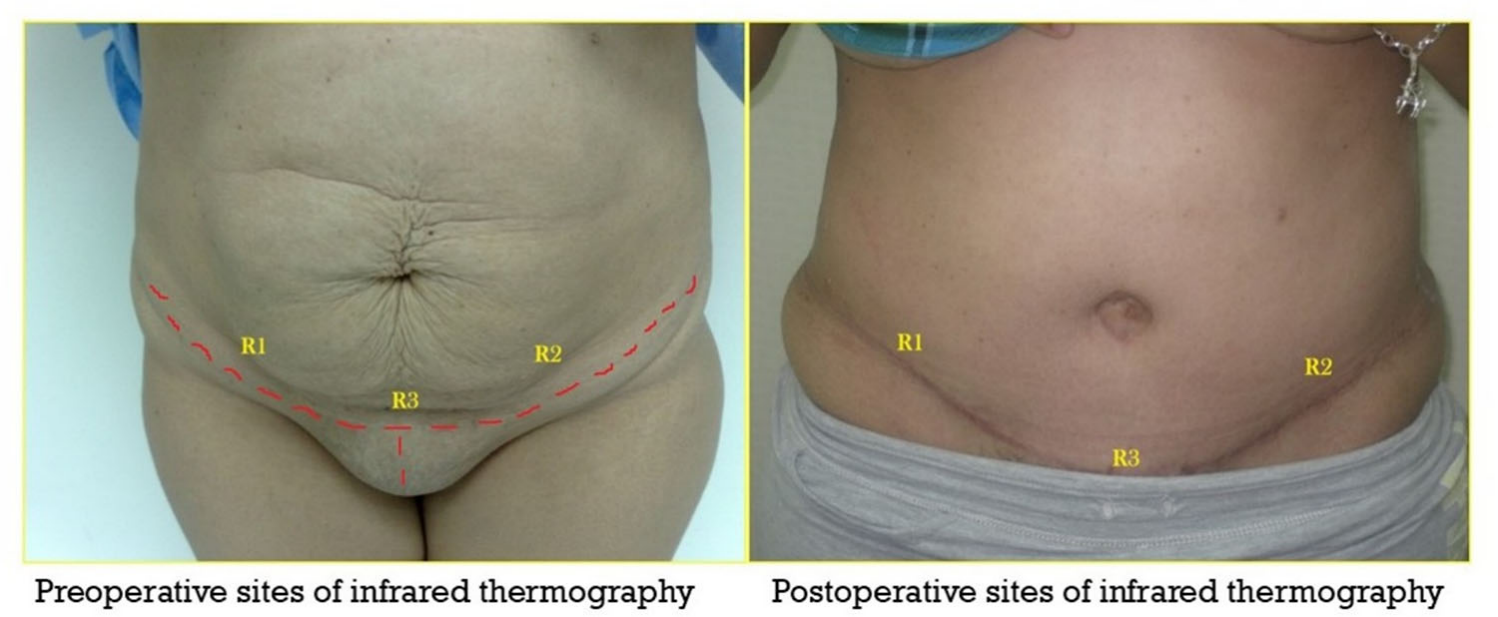
Pre- and postoperative sites of thermography measurements

The same three measurements were taken intraoperatively just after accomplishment of abdominoplasty and one month postoperatively. *R*1 and *R*2 were located at 10 cm from the midline on both sides just above the lower abdominal incision, while *R*3 was located on the midline just above the abdominoplasty incision corresponding to the most distal area of abdominoplasty skin flap (Fig. 1).

#### Equipment

The equipment used for the collection of thermal data consisted of a thermographic camera FLIR E60 SC (FLIR Systems, Wilsonville, Oregon, USA), with the following specifications: focal plane array of 320 × 240, Noise Equivalent Temperature Difference (NETD) of<  50 mK at 30 °C, the accuracy of± 2% of the overall temperature reading, long-wavelength (7–13.5 µm) and a 25º lens. It offers a temperature range of − 20–650 °C (− 4 to 1202°F) with an accuracy of± 2% and a thermal sensitivity of<  0.05 °C [25, 31, 32].

## Statistical Methods

Data were analyzed using IBM© SPSS© Statistics for Windows© version 27 (IBM Corp., Armonk, NY, 2020). Normality of numerical data distribution was examined using the Shapiro–Wilk test. Numerical data are presented as mean and standard deviation (SD) or mean and standard error (SE), and intergroup differences are compared using the unpaired t test. Categorical data are present as count and percentages and compared using Fisher’s exact test.

A generalized estimating equations (GEE) approach was used to model repeated measures data, accounting for the correlation of observations within subjects over time. The GEE model specified a normal distribution with an identity link function. An independent working correlation matrix was used to account for the repeated measures within subjects and robust standard errors were applied. Statistical significance was set at p < 0.05 (two-tailed).

## Results

The demographic data of the patients are summarized in Table 1. Concerning the age of the patients and BMI, there was no statistically significant difference between the two study groups (*p* > 0.05).

**Table 1:** Patients’ characteristics and operative data

Variable	Classic LAP (*N *= 10)	LAP with SF preservation (*N *= 10)	*P* value*
Age (yr)	39 ± 5	39 ± 5	.928
Body mass index (kg/m2)	31.3 ± 1.4	32 ± 1.4	.245
Preoperative hemoglobin (g/dl)	11.3 ± 0.8	11.7 ± 0.6	.258
Postoperative hemoglobin (g/dl)	9.8 ± 1.0	10.0 ± 0.9	.534
Absolute drop in hemoglobin (g/dl)	1.6 ± 0.4	1.7 ± 0.6	.640
Relative drop in hemoglobin (% of preoperative level)	14.0 ± 4.0	14.4 ± 5.4	.838
Tumescent fluid volume (ml)	1255 ± 236	1270 ± 241	.890
Fat aspirate volume (ml)	1505 ± 270	1365 ± 269	.261
Volume in drains on PO Day 1 (ml)	285 ± 37	281 ± 37	.813
Volume in drains on PO Day 2 (ml)	181 ± 37	190 ± 31	.562
Postoperative hospital stay			.141†
Two days	5 (50.0%)	9 (90.0%)	
Three days	5 (50.0%)	1 (10.0%)	

Data are mean ± standard deviation or count (percentage)

^*^. Independent-samples *t* test unless otherwise indicated

^†^. Fisher’s exact test

*PO* postoperative; *LAP* lipoabdominoplasty

**Fig. 2:**
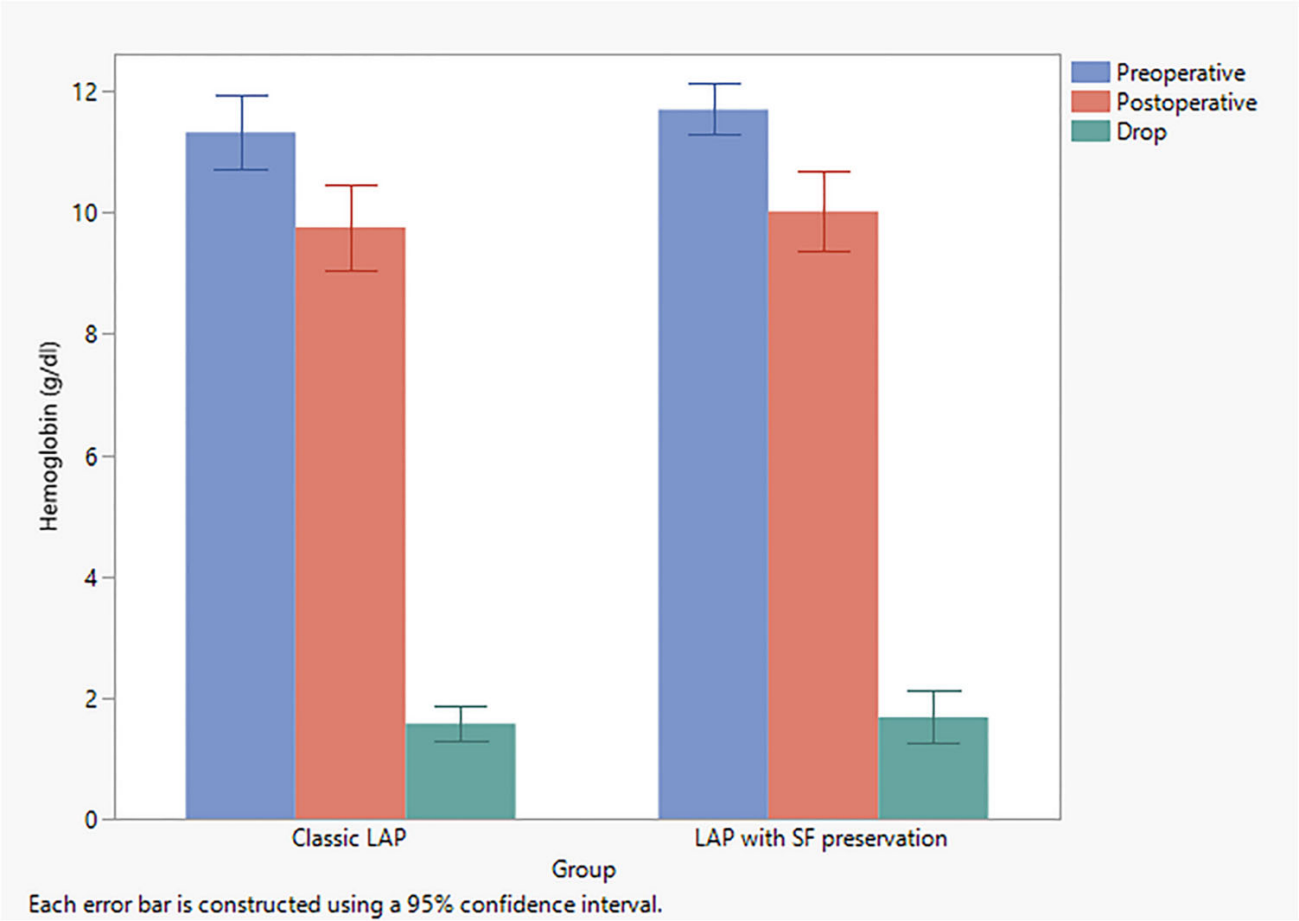
Perioperative change in hemoglobin level in both study groups. Error bars represent the 95% confidence interval (95% CI). *LAP* lipoabdominoplasty, *SF* scarpa preservation

Hemoglobin level was measured 48 hours postoperatively and was compared to the preoperative measurement. There was no statistically significant difference between the two measurements in both groups (*p* > 0.05). All patients did not have a postoperative blood transfusion (Table 2 and Fig. 2).

**Table 2:** Pre- and 48 hours postoperative levels of hemoglobin in both groups

Study groups	Preoperative HB level (g/dL)	48 hours Postoperative Hb levels (g/dL)	*P*. value
Group I (classic LAP)	11.7 ± 1.08 SD	11.8 ± 1.03 SD	> 0.05
Group II (LAP with SP)	9.8 ± 1.03 SD	9.9. ± 1.4 SD	> 0.05

**Fig. 3:**
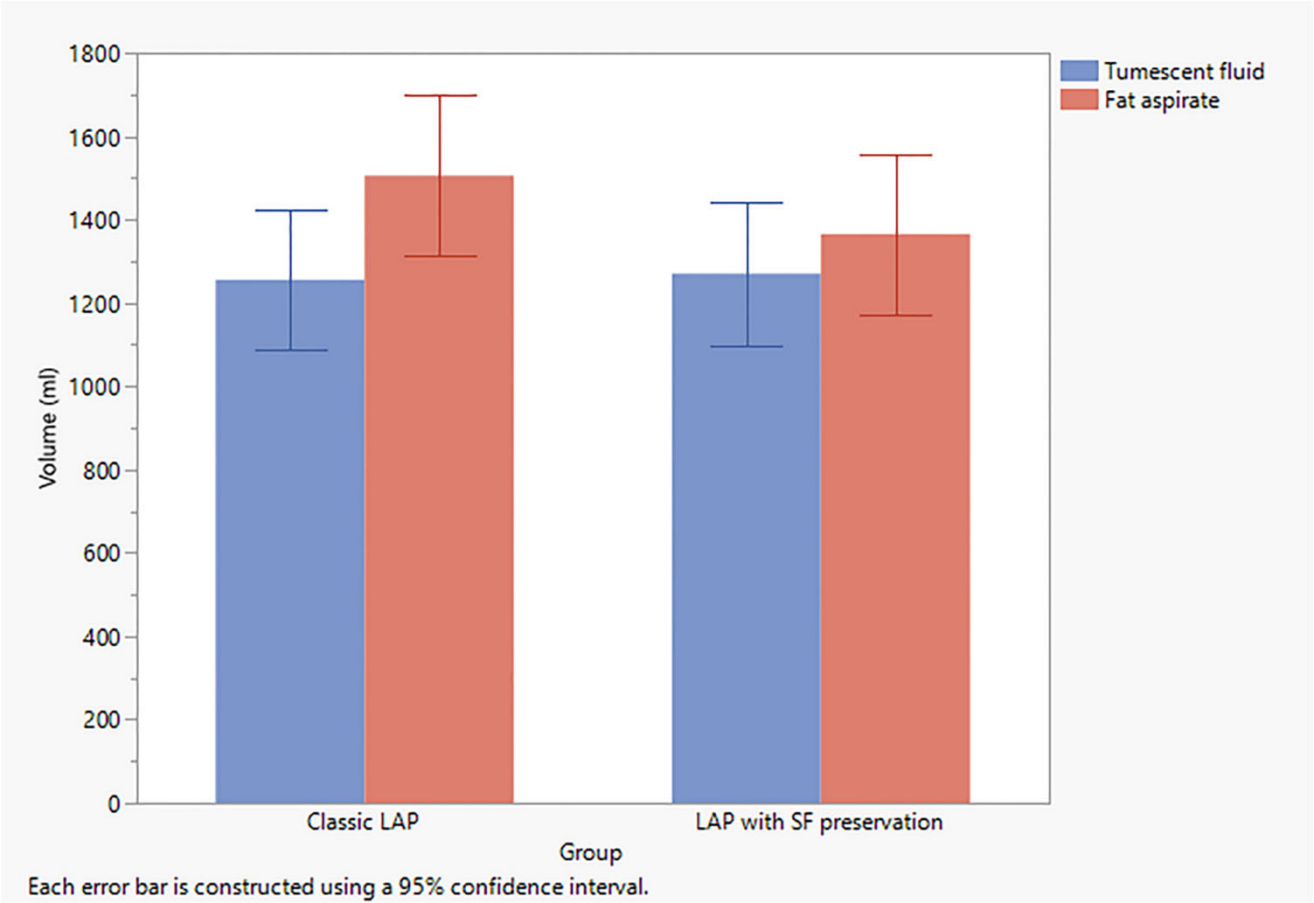
Tumescent fluid volume and volume of fat aspirate in both study groups. Error bars represent the 95% confidence interval (95% CI). *LAP* lipoabdominoplasty, *SF* scarpa preservation

The volume of fat aspirated was measured at the end of the surgery, giving enough time (120–150 minutes) to decant the fat and getting rid of the associated fluid and blood. The mean values of the volume of aspirated fat were statistically non-significant (*p* > 0.05) (Table 3 and Fig. 3).

**Table 3:** Mean values of the volume of aspirated fat in both groups

Study groups	Mean value of the volume of aspirated fat (in cc)	*P*. value
Group I (standard LAP)	1500 cc ± 25.53	> 0.05
Group II (LAP with SP)	1350 cc ± 36.83

**Fig. 4:**
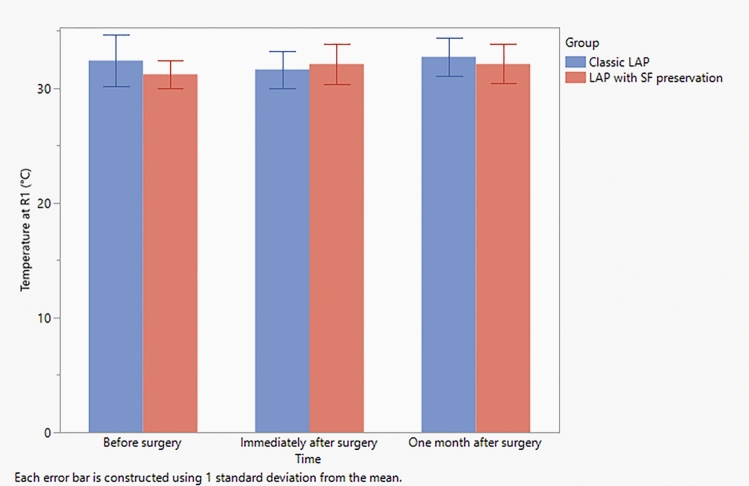
Observed temperature as recorded from *R*1 at each time point in both study groups. Error bars represent the standard deviation (SD)

**Fig. 5:**
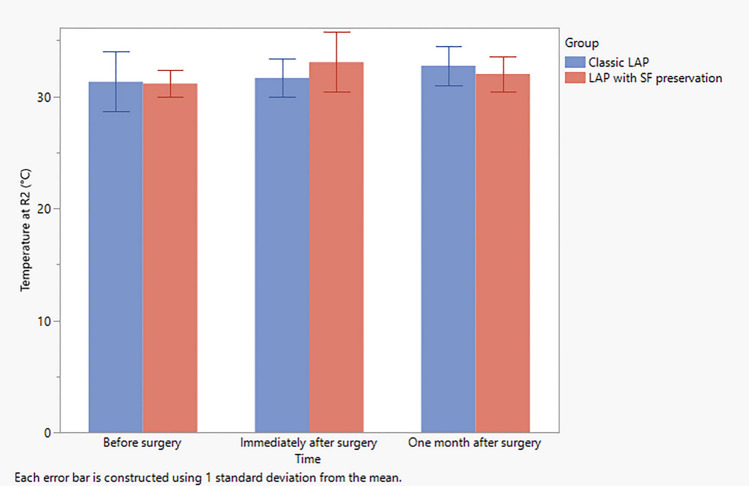
Observed temperature as recorded from *R*2 at each time point in both groups. Error bars represent the standard deviation (SD)

**Fig. 6:**
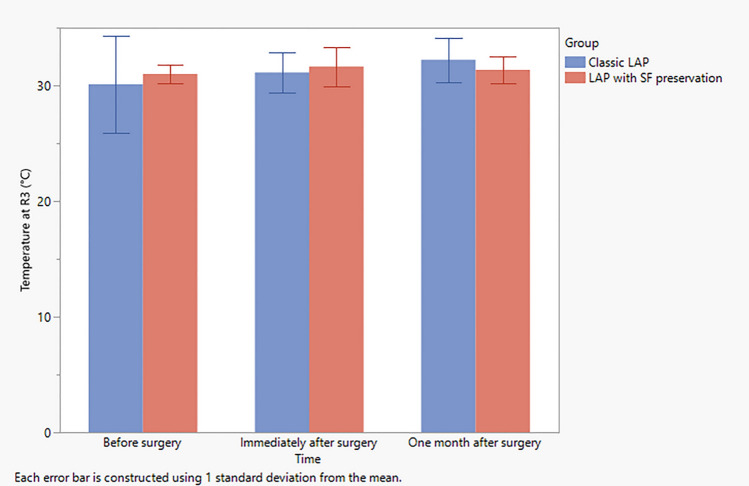
Observed temperature as recorded from *R*3 at each time point in both groups. Error bars represent the standard deviation (SD)

**Fig. 7:**
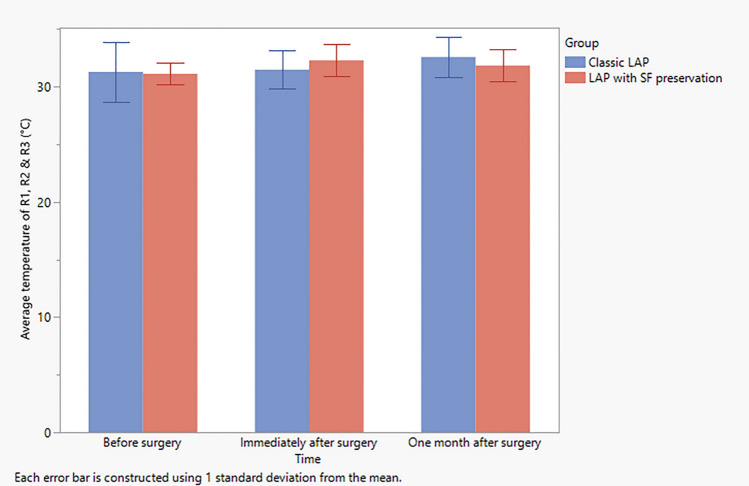
Observed temperature at each time point as recorded and averaged from *R*1, *R*2 and *R*3 in both groups. Error bars represent the standard deviation (SD)

The preoperative infrared thermography measurement of the regions of interests in standard lipoabdominoplasty group revealed mean values of 32.4 ± 2.2, 31.3 ± 2.7, 30.1 ± 4.2 at *R*1, *R*2, and *R*3, respectively, while those of the scarpa fascia preservation group revealed mean values of 31.2 ± 1.2, 31.2 ± 1.2, and 31.0 ± 0.8 for *R*1, *R*2 and *R*3, respectively (Table 4). However, one month postoperatively, the infrared thermography measurement revealed mean values of 32.8 ± 1.7, 32.8 ± 1.8, and 32.2 ± 1,9 for *R*1, *R*2 and *R*3, respectively, for the standard lipoabdominoplasty group, while those of the scarpa fascia preservation group revealed mean values of 32.1 ± 1.7, 32.0 ± 1.5, and 31.4 ± 1,1 for *R*1, *R*2, and *R*3, respectively (Table 4 and Figs. 4, 5, 6 and 7).

**Table 4:** Observed change in temperature at the three regions of interests in both groups

		Classic LAP (*N *= 10)		LAP with SF preservation	
Variable	Time	Mean	SD	Mean	SD
Temperature at *R*1 (°C)	Before surgery	32.4	2.2	31.2	1.2
	Immediately after surgery	31.6	1.6	32.1	1.7
	One month after surgery	32.8	1.7	32.1	1.7
Temperature at *R*2 (°C)	Before surgery	31.3	2.7	31.2	1.2
	Immediately after surgery	31.7	1.7	33.1	2.7
	One month after surgery	32.8	1.8	32.0	1.5
Temperature at *R*3 (°C)	Before surgery	30.1	4.2	31.0	0.8
	Immediately after surgery	31.1	1.8	31.6	1.7
	One month after surgery	32.2	1.9	31.4	1.1
Average temperature of *R*1, *R*2, and *R*3 (°C)	Before surgery	31.3	2.6	31.1	0.9
	Immediately after surgery	31.5	1.6	32.3	1.4
	One month after surgery	32.6	1.8	31.8	1.4

*SD* Standard deviation

Based on the statistical analyses, preservation of scarpa fascia in lipoabdominoplasty does not seem to enhance the vascular perfusion of the abdominal skin after abdominoplasty. Indeed, preservation of the epigastric and subcostal perforators may play a major role in the augmentation of distal skin perfusion.

## Discussion

Abdominoplasty is one of the most popular and most performed aesthetic surgical procedures [1, 2]. It is frequently associated with complications which can be preventable by respecting the vascular anatomy of the abdominal skin and proper monitoring of the skin perfusion in the early postoperative time [33, 34]. One of the main risks of abdominoplasty is delayed wound healing and/ or less than optimum perfusion at the most distal part of abdominal skin, which can give a negative impact on the aesthetic outcome, leading to emotional stress to the patient, and increase health care costs [34–36].

Abdominoplasty requires undermining of a large skin–fat flap that has a particular pattern of blood supply [37]. Complications like wound dehiscence, skin necrosis, and wound infection can be due to a decreased perfusion of this flap. As adequate blood perfusion is crucial for normal wound healing, a better understanding of abdominal skin perfusion after abdominoplasty may contribute to reducing wound healing problems. Furthermore, a reliable method of skin flap monitoring in the early postoperative time can help in the early diagnosis of low perfusion and necessary measures that can be undertaken to salvage the flap.

Infra-red thermography (IR) measurement of skin surface temperature provides indirect information on skin perfusion. Studies have shown a good correlation between thermographic results and laser Doppler flowmetry, and between thermographic results and skin perfusion monitored with indocyanine green fluorescence video angiography and isotope perfusion [28, 38–41]. Dynamic infrared thermography (DIRT) is based on the relationship between skin perfusion and the change in the rate and pattern of skin surface temperature following a transient thermal challenge [42]. Its Role in monitoring flap perfusion and in preoperative perforator mapping has been reported in a number of studies [42–44].

The purpose of the present study was to determine the effect of preserving the scarpa fascia in lipoabdominoplasty on the postoperative abdominal skin perfusion as monitored with infrared thermography. Valença-Filipe and his coworkers in 2023 investigated this hypothesis on a total of 12 women divided randomly into two groups (Classic) and (scarpa- sparing) abdominoplasty. They found that an improved abdominal skin vascularization when scarpa fascia is preserved [25]. However, in the present study, 20 women seeking for lipoabdominoplasty were randomly divided into two groups, standard (group I) and lipoabdominoplasty with preservation of the scarpa fascia (Group II).

However, the difference in measurements of the infrared thermography (IR) for the chosen areas or the regions of interests (RIO) were statistically non-significant in the pre, immediate postoperative, and one-month postoperative.

Valença-Filipe and his coworkers in 2023 justified their result based on several theories [25]. The abdominal wall has two fat compartments; the superficial and the deep fat compartments which differ in their physical properties [45, 46]. The deep fat is more flexible than the superficial fat; consequently, the former achieves a higher lateral displacement. Ultimately, preserving Scarpa fascia and the deep fat may create a “stickier” interface between tissues, resulting in better resistance to shearing movements, tissue adhesion, and healing. Another theory refers to lymphatics’ preservation. According to this theory, when preserving Scarpa fascia and its deep tissues, the lymphatic drainage would be maintained as the lymphatic vessels would be kept along with their connection to inguinal lymph nodes, limiting fluid accumulation between surfaces, reducing dead space, and decreasing seroma rate [17]. The third explanation refers to better vascularization when preserving scarpa fascia and the underlying deep fat, respecting the normal physiology of the abdominal tissues. Several studies have been conducted to better understand the abdominal wall blood supply [47–49]. It has been shown that the superficial inferior epigastric artery travels just superficially to the Scarpa fascia and releases small branches to a fascial vascular network contained within the fascia [47]. In addition, the deep inferior epigastric vessels and their perforators may improve vascularization when performing a scarpa-sparing abdominoplasty. However, the study of Valença-Filipe and his coworkers in 2023 had a limitation of a small sample size (6 patients in each group) but a relatively more number in the current study (10 patients on each group) [25].

In the current study, three points of references were chosen to measure the temperature using the infrared thermography camera. These points were corresponded to the distal areas of abdominal skin after accomplishing the lipoabdominoplasty. The preoperative measurements in both groups were statistically non-significant (*p* > 0.05). The surgical technique was standardized, and all the procedures were performed by the senior authors. The measurements of the immediate postoperative and one month after surgery were statistically non-significant difference (*p* > 0.05). According to the results of the current study, preservation of the scarpa fascia did not appear to enhance the perfusion of the abdominal skin after lipoabdominoplasty.

The justification of this results may be the fact that vascular perfusion of the abdominal skin greatly depends on the preservation of as much as vascular perforators supplying the skin [50, 51]. In contrary, the extent of the undermining and stretching on the abdominal skin during closure represents the main factors controlling the perfusion of the skin, particularly the central zone just overlying the abdominoplasty incision line. Preservation of scarpa fascia in lipoabdominoplasty seems to reduce the development of seroma due to preservation of lymphatics, reducing the surgical dead space, and preventing the shearing force between two non-identical types of tissue [17, 20, 23]. Tourani and his colleagues in 2015 stated that the decrease in the incidence of postoperative seroma formation is not due preserving the lymphatics with the fascial planes but due to the elimination of the postoperative shearing forces which enhances rapid wound healing [18]. Razzano et al. [52] commented on the study of Tourani et al, explaining that keeping a deep layer of fat over the rectus sheath maintained the deep lymphatics with subsequent decreased formation of seroma. [52]. Tourani et al. [53] replied to their comment stressing on the main reason of seroma formation is the shearing forces rather than the lymphatic drainage [53].

However, the primary objective of the current study was to investigate the effect of preserving scarpa fascia in lipoabdominoplasty on the perfusion of abdominal skin.

## Conclusion

Scarpa fascia preservation contributed to decrease the formation of post lipoabdominoplasty seroma formation. Objective measurement using infrared thermography (IRT) of the distal skin perfusion after scarpa fascia preservation in lipoabdominoplasty did not enhance skin vascularity and perfusion. Decreased dead space, and elimination of seroma formation may contribute to better wound healing with subsequent better skin perfusion.

- The authors declare that they have no conflicts of interest to disclose.
- The study has been approved by the national research ethics committee and has been performed in accordance with the ethical standards as laid down in the 1964 Declaration of Helsinki and its later amendments or comparable ethical standards.
- For this type of study informed consent is not required
